# The effect of fatigue on postural control in individuals with multiple sclerosis: a systematic review

**DOI:** 10.1186/s12883-023-03464-4

**Published:** 2023-11-17

**Authors:** Parisa Sedaghati, Mohammad Alghosi, Freshteh Hosseini

**Affiliations:** Department of Sports Injury and Corrective Exercise, Faculty of Physical Education & Sport Sciences, University of Guilan, Rasht, Iran

**Keywords:** Demyelinating Diseases, Disseminated sclerosis, Balance, Exhaustion, Walk test

## Abstract

**Background:**

Fatigue is the most disabling symptom for individuals with multiple sclerosis (MS), which can significantly affect postural control (PC) by impairing the ability of the central nervous system to modulate sensory inputs and coordinate motor responses. This systematic review aimed to investigate the effect of fatigue on PC in individuals with MS..

**Methods:**

This systematic review is reported according to the Preferred Reporting Items for Systematic Reviews and Meta-Analyses (PRISMA) guideline and registered in PROSPERO with ID CRD42022376262. A systematic search was performed in the Web of Science, PubMed, Scopus, and Google Scholar until January 2023, and a manual search was performed using the reference lists of included studies. Two authors independently selected the studies, extracted data, and evaluated their methodological quality using the Downs and Black checklist. The process was later discussed with a third author..

**Results:**

Five studies were included in this review, of which consistent evidence investigating a direct relationship between fatigue and PC in individuals with MS. All the studies reported negative effects on PC. Four studies employed walking tests as their primary protocol for inducing fatigue, while one study implemented a strength testing protocol for both legs, serving as a fatigue-inducing activity.

**Conclusions:**

The evidence suggests that individuals with MS may experience PC deficits due to fatigue. However, the present body of literature exhibits limitations regarding its quality and methodology. Gender differences, balance, fatigue task, and muscle function are essential factors that need to be considered when investigating the relationship between fatigue and PC deficits in MS. Further high-quality research is necessary to comprehend the complex interplay between MS-related fatigue and PC deficits after physical activity.

## Background

Multiple sclerosis (MS) is the most prevalent progressive and chronic disabling neurologic disease that affects the central nervous system (CNS) through demyelination, inflammation, and axonal loss [1, 2]. In 2020, MS was estimated to affect up to 2.8 million people worldwide with a significant impact on physical, emotional, social, and cognitive functioning [3, 4]. MS exhibits varying symptoms depending on the affected region, encompassing cerebellar, motor, sensory, emotional, and sexual manifestations [5]. Among the many symptoms associated with MS, fatigue has been identified as a significant concern, with up to 50–60% of patients experiencing this symptom [5]. In addition to the limitation in MS individuals’ activities of daily living and social lives from fatigue, it also hurts cognitive functions, decreasing attention and concentration [6]. In some disorders like MS, fatigue may be associated with motor and, or mood disorders, so it is challenging and sometimes impossible to determine whether fatigue is an aspect of these features or a symptom [7]. Fatigue physiologically is defined as “the inability of a muscle or group of muscles to sustain the required or expected force” by Bigland-Ritchie et al. [8]. Fatigue may occur from failure at force-generating capacity within the muscle itself (peripheral fatigue), or because of a disability to maintain the central drive to spinal motor neurons (central fatigue) [7].

Also, impaired balance is typically the primary symptom of MS, and it arises from a combination of slowed somatosensory conduction and impaired central integration [9] which cause abnormal gait control, and many fall frequently [10–14]. In this aspect, MS can potentially impact the entire CNS, resulting in various impairments in neurological functions [11]. Integrating various sensorimotor modalities, including visual, vestibular, and proprioceptive information, plays a significant role in postural control (PC) and sustaining an upright stance [15, 16]. These complex sensorimotor processes contribute to regulating body sway and facilitate coordinated movement patterns that maintain the center of mass within the limits of stability. Therefore, Understanding how different sensory inputs interact and contribute to PC is essential to developing interventions that can improve balance and prevent falls in vulnerable populations [15, 16]. Due to the presence of impairments in multiple processes, individuals with MS tend to exhibit weaker PC, as indicated by greater amounts of postural sway when compared to healthy controls [11, 12, 17, 18].

According to a recent systematic review study, individuals with MS experienced a positive impact on their fatigue levels as a result of sensory integration-based interventions. This led to improved balance and an overall increase in their quality of life. These findings may have important implications for managing symptoms and improving outcomes for individuals with MS [19]. Also, brain structural and functional alterations are seen in MS-related fatigue [20]. Particularly, sensorimotor network impairment and abnormal activation of the thalamus are associated with fatigue.

To date, no systematic review has synthesized the available data on the effect of fatigue on the PC of individuals with MS. By providing a comprehensive evaluation of existing research, this review aimed to address this knowledge gap and shed light on the relationship between fatigue and PC in this population.

## Methods

This systematic review is reported according to the Preferred Reporting Items for Systematic Reviews and Meta-Analyses (PRISMA) guideline [21] and was registered in the International Prospective Register of Systematic Reviews (PROSPERO) with registration number: CRD42022376262.

### Search strategy

The relevant studies were identified through a systematic computerized search in Web of Science, PubMed, Scopus, and Google Scholar from 1st January 1974 until 1st January 2023. Two authors independently (M.A. and F.H.) searched four electronic databases. Any disagreement between the two authors was resolved by discussion and a third author’s opinion (P.S.) until a consensus was reached. The search strategy included MeSH terms and text words for a comprehensive search. Three sets of entry strings were mixed with ‘AND’ to produce the following syntax: (“postur*” OR “postural control” OR “postural sway” OR “postural stability” OR "postural steadiness” OR balance OR equilibrium OR sway) AND (fatig* OR lassitude OR exhaustion) AND (“multiple sclerosis”). Restrictions applied were human studies and full-text articles. Also, the reference lists of included studies screening and a grey literature search were performed to identify additional eligible studies. Retrieved studies were transferred into Endnote, and duplicates were deleted. The specific search strategy for each database is presented in Table 1.

**Table 1:** Search strategy

Database	Search string
Web of Science	(“postur*” OR “postural control” OR “postural sway” OR “postural stability” OR "postural steadiness” OR balance OR equilibrium OR sway) AND (fatig* OR lassitude OR exhaustion) AND (“multiple sclerosis”)
PubMed	(“postural control“[Title/Abstract] OR “postural sway“[Title/Abstract] OR "postural stability“[Title/Abstract] OR “postural steadiness“[Title/Abstract] OR balance[Title/Abstract] OR equilibrium[Title/Abstract] OR sway[Title/Abstract]) AND (fatigue[Title/Abstract] OR lassitude[Title/Abstract] OR exhaustion[Title/Abstract]) AND (“multiple sclerosis“[Title/Abstract]) AND ((fft[Filter]) AND (humans[Filter]))
Scopus	TITLE-ABS-KEY(“postur*” OR “postural control” OR “postural sway” OR “postural stability” OR “postural steadiness” OR balance OR equilibrium OR sway) AND TITLE-ABS-KEY(fatig* OR lassitude OR exhaustion) AND TITLE-ABS-KEY(“multiple sclerosis”)

### Eligibility criteria and screening methods

Following the search process, two authors (M.A. and F.H.) independently screened all titles and abstracts generated by the search procedure. Studies were selected according to the inclusion and exclusion criteria based on Population, Intervention, Comparison, and Outcome (PICO framework) [22] (Table 2).

**Table 2:** Eligibility criteria based on PICO

	Inclusion criteria	Exclusion criteria
Population	• Individuals with multiple sclerosis • Individuals aged from 18 to 65 years • Any sex	• Any surgery affecting the lower extremities or the spine in clinical history
Intervention	• Prolonged and/or exhaustive activity organized to lead to fatigue	• No postural control measured
Comparison	• Passive control groups or conditions	• Did not include a group or conditions as comparators for quantifying fatigue following the intervention
Outcome	• Studies investigating a relationship between fatigue and postural control (must include one of the following measures: center of pressure, the center of gravity, the center of mass, and other functional scales of postural control).	• Studies not investigating a direct relationship between fatigue and postural control

### Data extraction

The two reviewers, M.A. and F.H., independently extracted data from eligible studies, if available, and verified it with another reviewer, P.S. The study details, including the author, year of publication, type of study, demographic variables of the participants such as sample size, age, gender, MS type, disability level, and disease duration, baseline therapies or pharmacological interventions, fatigue protocol, fatigue evaluation, outcome measures, measurement position, main outcomes, and conclusion were extracted. If some necessary information was not provided in the paper, corresponding authors were contacted via email up to three times to obtain the requested data.

### Quality assessment

Since this review included different types of articles, two authors (M.A. and F.H.) independently assessed the quality of evidence of the studies using the criteria proposed by Downs and Black checklist [23], as this checklist is the best option to evaluate the quality and risk of bias for both randomized and non-randomized controlled trials [24]. The checklist included 27 questions grouped into five categories, including reporting (10 items), external validity (3 items), bias (7 items), confounding (6 items), and power (1 item). The power item (item 27) was simplified to a binary score based on whether or not a sample size calculation was performed. A score of 1 indicates the presence of a sample size calculation, while a score of 0 indicates its absence. With this alternation, the best possible score is 28. The quality of induced studies consists of the following score ranges: excellent [26–28], good [20–25], fair [15–19], and poor (≤ 14). This modification was done in previous reviews related to MS [19, 25]. The opinion of a third author (P.S.) was taken if a disagreement arose.

### Data synthesis

A narrative data synthesis was performed. For complete reporting and transparency in the manuscript, this systematic review followed the PRISMA statement [21], and due to the different fatigue protocols and outcomes, meta-analysis was impossible. This was conducted by Synthesis Without Meta-analysis (SWiM) reporting guideline [26].

## Results

### Study identification

**Fig. 1:**
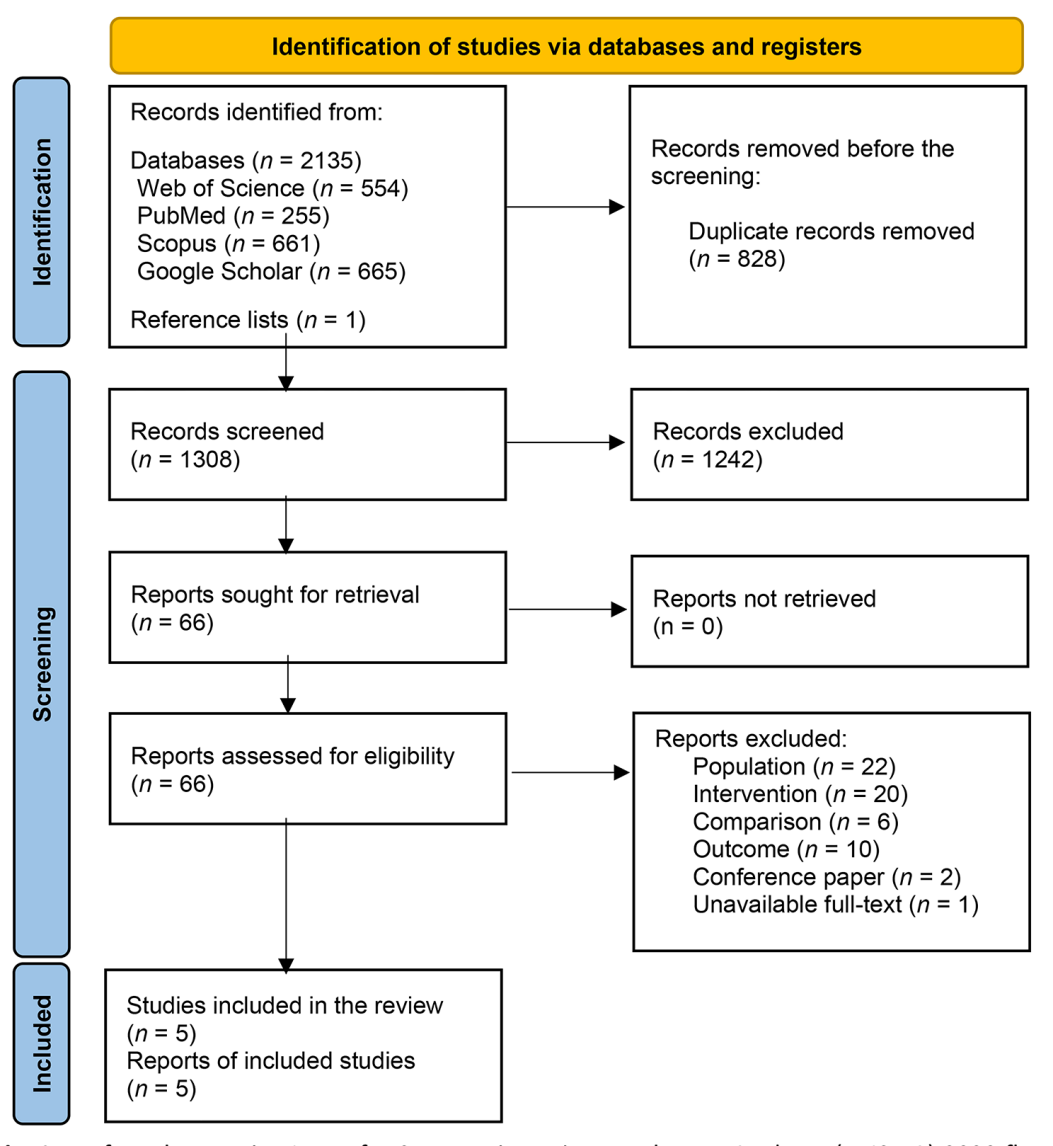
Preferred Reporting Items for Systematic Reviews and Meta-Analyses (PRISMA) 2020 flow diagram for new systematic reviews, including searches of databases and registers

The electronic databases search process is presented as a flow diagram (Fig. 1) based on the PRISMA guideline [21]. The search strategy retrieved 2136 articles through manual search. After removing duplicates, 1308 studies remained for further screening according to the inclusion and exclusion criteria. Only five studies successfully met eligibility criteria and entered the quality assessment phase.

### Study characteristics

All reviewed articles were published between 2010 and 2023 and written in English. One hundred twenty individuals participated in these studies. Three out of five studies [27–29] were performed without a comparison group [28, 30, 31]. In four studies [27–30], both sexes were included, whereas in one study [31], only female individuals were included. The mean age of the participants was 46.6 years (SD 9.9), and 35% were male [28, 30, 31, 33, 34]. The Six-Minute Walk Test (6MWT) was used in four of the studies [27–30], while one study [31] used strength testing for both legs. Additionally [34, 39], Table 3 summarized the specific characteristics and retrieved data of all five studies.

**Table 3:** Characteristics of included studies

Study	Type of Study	Participant demographics	Baseline therapies or pharmacological intervention	Fatigue protocol	Fatigue evaluation	Outcome measures	Measurement position	Main outcomes	Conclusion
Drebinger et al. (2020)	Prospective observational	N: 43 (Women = 20, Men = 23) Age: 48.6 (13.7) MS type: NA EDSS: 1–6 Disease duration: NA	Disease-modifying therapies (12), antidepressants (3), fampridine (2), other CNS medication (8), and other non-CNS medication (15)	6MWT	Fatigue scale for motor and cognitive functions questionnaire	Sway closed stance eyes open angular speed (degrees/s), Sway closed stance eyes closed angular speed (degrees/s), Romberg ratio angular speed (-)	Static balance function performance in eyes open and eyes closed conditions	Postural sway with closed eyes (*P* < 0.05) Postural sway with open eyes (*P* < 0.05) Romberg ratio (*P* > 0.05)	Postural sway will increase after 6 min of walking.
Jallouli et al. (2022)	Quasi-experimental	N: 18 (Women = 11, Men = 7) Age: 30.7 (8.2) MS type: RRMS EDSS: 0–4 Disease duration: 5 years	NA	1) 3 sets of 5-repetition sit-to-stand test 2) 6MWT 3) 3 sets of 5-repetition sit-to-stand test	Visual analogue scale of fatigue	Mean COP velocity (mm/s), COP sway area (mm^2^)	Bipedal and unit pedal postural balance performance in eyes open and eyes closed conditions	COP velocity (*P* < 0.01) COP sway area (*P* < 0.01)	Fatiguing tasks negatively affected postural control.
Sanni et al. (2021)	Quasi-experimental	Number: 6 (Women = 3, Men = 3) Age: 47.4 (NA) MS type: RRMS and PPMS PDDS: 3–5 Disease duration: 5 years	NA	6MWT	Visual analogue scale of fatigue	COP ellipse sway area with eyes closed and eyes open (mm^2^)	Static balance with eyes closed and eyes open	69% increase in the COP ellipse sway area with eyes opened (Cohen’s D 0.5) 20% increase in the COP ellipse sway area with eyes closed (Cohen’s D 0.2)	Six minutes of self-paced walking worsened balance. among people with MS.
Karpatkin et al. (2013)	Quasi-experimental	N: 29 (Women = 20, Men = 9) Age: 52.0 (8.8) MS type: RRMS, SPMS, and PPMS EDSS: 1.5-6 Disease duration: 14 years	Antispasticity medication: 12 individuals Antifatigue medication: 9 individuals	6MWT	Fatigue severity scale	Berg balance scale score (-)	Static balance with eyes open	BBS (*P* < 0.001)	Fatigue alters the performance of people with MS on the BBS.
Emmerik et al. (2010)	Quasi-experimental	N: 24 (Women = 24, Men = 0) Age: 53.9 (8.9) MS type: RRMS, SPMS, and PPMS EDSS: 2–6 Disease duration: 15 years	MS group: MS drugs (9), anti-depressants (5), anti-fatigue (3), anti-spasticity (3) and anti-convulsive/sedative (4)	Strength testing for both legs (Peak knee extensor and dorsiflexor isometric torque and isotonic power)	Visual analogue scale of fatigue	Loading asymmetry (%), medial-lateral COP displacement (mm), anterior-posterior COP displacement (mm)	Static (with eyes open and eyes closed) and dynamic balance	COP loading asymmetry (*P* > 0.05) COP anterior displacement (*P* < 0.05) COP posterior displacement (*P* > 0.05) COP lateral displacement (*P* > 0.05)	Additional fatigue in the MS group did affect postural control in the more challenging balance conditions.

**Abbreviations**: MS: multiple sclerosis, EDSS: expanded disability status scale, PDSS: patient determined disease steps, CNS: central nervous system, COP: center of pressure, mm: millimeter, S: second, BBS: berg balance scale, six-minute walk test: 6MWT, relapsing-remitting MS: RRMS, secondary progressive MS: SPMS, primary progressive MS: PPMS, N: number, NA: not applicable

### Outcomes

All the studies included in the review [27–31, 27–29, 32, 34] reported negative effects on PC. Four studies [27–30] executed similar fatigue protocols (walking test), and one study [31] used a strength testing protocol for both legs, which served as a fatigue-inducing activity [32]. Th [36]e 6MWT is not specifically designed to measure fatigue, it indirectly provides information about fatigue-related factors during the test, such as gait speed [33]. Studies have shown that individuals with MS experience motor fatigue in both 6MWT distance and speed when compared to healthy controls [34]. The following procedures for fatigue for PC assessment in four studies [27, 28, 30, 31] were performed with eyes closed and eyes open. Drebinger et al. [30] explored fatigue effects on PC using visual perceptive computing. Jallouli et al. [27] examined the effect of a fatiguing task by using a stabilometric platform. Sanni et al. [28] evaluated the relationship between fatigue and following balance assessment with a force plate. Karpatkin et al. [29] investigated a direct relationship between fatigue and PC by performing the Berg balance scale test. Emmerik et al. [31] investigated the changes in balance at different levels of self-reported fatigue.

### Quality assessment

The quality assessment results ranged from 13 to 16 (Mean: 14.8), presented in Table 4. Perfect agreement between authors across the 27 items with the Cohen’s kappa value 1 (0-0.20, poor agreement; 0.21–0.40, fair agreement; 0.41–0.60, moderate agreement; 0.61–0.80, good agreement; and 0.81-1.00, perfect agreement) [35] was obtained. All studies reported the aims, main outcome measures, participant characteristics, and probability values. Regarding the external validity, bias, and confounding items of the checklist, studies didn’t provide any information on the participants blinding to intervention, outcome assessors blinding, analysis adjusts for different lengths of follow-up of participants, allocation concealment, adjustment for confounding variables in the main analysis and adjustment for loss to follow-up in the main analysis. Only, one study reported sample size calculation [27].

**Table 4:** Quality assessment of included studies

Article	Downs and Black checklist item																												Total (0–28)
	Reporting										External validity			Bias							Confounding						Power		
	1	2	3	4	5	6	7	8	9	10	11	12	13	14	15	16	17	18	19	20	21	22	23	24	25	26	27		
Drebinger et al. (2020)	1	1	1	1	2	1	1	0	0	1	1	1	0	0	0	1	0	1	1	1	1	0	0	0	0	0	0	16	
Jallouli et al. (2022)	1	1	1	1	1	1	1	0	0	1	1	1	0	0	0	1	0	1	1	1	1	0	0	0	0	0	1	16	
Sanni et al. (2021)	1	1	1	1	1	1	0	0	0	1	0	1	0	0	0	1	0	1	1	1	1	0	0	0	0	0	0	13	
Karpatkin et al. (2013)	1	1	1	1	1	1	0	0	0	1	0	1	1	0	0	1	0	1	1	1	1	0	1	0	0	0	0	15	
Emmerik et al. (2010)	1	1	1	0	1	1	1	0	0	1	0	1	0	0	0	1	0	1	1	1	0	1	1	0	0	0	0	14	

**Notes**: 2, criterion fully met (item 5); 1, criterion met or partially met (item 5); 0, criterion not met

Downs and Black checklist item descriptions: 1, hypothesis/aims/objectives reported; 2, main outcome measures reported; 3, participant characteristics reported; 4, intervention details reported; 5, principal confounders reported; 6, main findings reported; 7, variability in main outcomes reported; 8, adverse events reported; 9, loss to follow-up reported; 10, probability values reported; 11, source population representative of entire population; 12, study population representative of source population; 13, study setting representative of usual care; 14, participants blinded to intervention; 15, outcome assessors blinded; 16, no retrospective subgroup analysis; 17, analysis adjusts for different lengths of follow-up of participants; 18, statistical tests are appropriate; 19, reliable compliance with intervention; 20, outcome measures are valid and reliable; 21, recruitment of study groups from same population; 22, recruitment of participants over same period;23, randomization of participants; 24, allocation concealment; 25, adjustment for confounding variables in main analysis; 26, adjustment for loss to follow-up in main analysis; 27, inclusion of sample size calculation

## Discussion

This systematic review aimed to investigate whether there is a relationship between fatigue and PC in individuals with MS. The current literature on the impact of fatigue on PC in individuals with MS is limited. Tasks that induce fatigue have been shown to negatively affect PC in individuals with MS [27–31]. This observation is consistent with previous research highlighting the negative impact of fatigue on various aspects of motor function in individuals with MS. There are several possible reasons for this finding which will be argued in the following paragraphs.

By reviewing the methodological quality of included studies, a large amount of lack of loss to follow-up was noted [27–31], and may not have distinguished all possible causes of missing follow-up data which entailed a risk of bias. Also, while this was an exploratory study, and we evaluated factors that predicted loss to follow-up, it is still possible that bias may have been introduced due to loss to follow-up. Additionally, it is essential to note that all of the included articles in our systematic review lacked blinding [27–31]. Lack of blinding in a study can introduce biases in various ways, depending on who remains unblinded among the study’s participants. Individuals assigned to the experimental group may have more positive expectations or report better outcomes to appease treatment providers. In contrast, those in the control group may have lower expectations and report poorer outcomes [36]. Thus, implementing blinding protocols where possible in research studies examining fatigue in MS can improve the quality and reliability of study results.

Fatigue severity in MS may depend on several clinical factors, including the number of years since onset, the specific MS subtype, the level of baseline disability, and the degree of disease activity. To address these factors, we compared the included studies in terms of the level of disability, MS subtype, and disease duration. The included studies revealed a wide range of disability levels, as measured by the Expanded Disability Status Scale (EDSS), with scores ranging from 1 to 6, 0–4, 3–5, 1.5-6, and 2–6 [27–31]. The comparison of PC in MS patients with EDSS scores less than 2.5, indicating minimal impairment in functional subsystems, with healthy groups, using criteria such as sway area, velocity, and displacement of the center of pressure, demonstrates that their ability to maintain PC is similar to that of healthy individuals [37]. However, as the degree of disability increases, significant differences are observed between the more severely affected MS patients and those with only mild or moderate disability [38]. Hence, a wide range of EDSS of included studies may be considered as a confusing factor regarding the expression and progression of fatigue related to the PC. Also, different types of MS, including relapsing-remitting MS, secondary progressive MS, and primary progressive MS [39], may play a role in the various results observed during PC evaluation with fatigue. In this regard, cognitive-postural interference was found to be more pronounced in SPMS patients, as they exhibited a higher dual-task cost compared to those with RRMS and healthy controls [40]. This indicates a greater impact on PC when comparing different types of MS.

Disability progression in MS appears to be linked to heightened fatigue and PC issues. Motl et al. [41] found that higher levels of disability were significantly correlated with increased fatigue in individuals with MS. Another study by Prosperini et al. [42] demonstrated that disability progression was associated with worsening PC, as measured by the Berg Balance Scale. These findings are consistent with those of Karpatkin et al. [29] whose study also employed the Berg balance scale score. Hence, it is crucial to manage disability progression to mitigate the impact on fatigue and PC problems in individuals with MS. While it is recommended that individuals with MS engage in regular physical activity, such as walking, to improve their quality of life, it is important to note that disability progression may still occur even in the absence of relapses. However, they often engage in less physical activity due to increased fatigue, mobility impairment, and fear of falling after a previous fall [28]. Furthermore, the included studies identified other factors that may contribute to PC impairment in individuals with MS, such as gender differences [27] and lower leg muscle function [28]. These factors should be considered when assessing PC in individuals with MS, as they may require different management strategies..

Accuracy and reliability of physician outcomes versus patient-reported outcomes is another important topic to consider. Physicians may not always be aware of the full extent of a patient’s symptoms, particularly if the patient does not report them or if the physician does not ask about them specifically [43]. Patient-reported outcomes provide a more direct measure of the patient’s experience of their symptoms and the impact of various interventions on their quality of life [43]. Patients may be more likely to report symptoms that are not easily observable by physicians, such as fatigue or cognitive difficulties [44]. However, patient-reported outcomes may also be influenced by factors such as mood, anxiety, or other comorbidities that could affect their perception of their symptoms [44]. In this regard, the Fatigue Scale for Motor and Cognitive Functions Questionnaire demonstrates high sensitivity and specificity in detecting fatigue in patients with MS (Cronbach’s alpha a > 0.91 and test–retest reliability r > 0.80 [45]. The Visual Analogue Scale of Fatigue exhibits a strong correlation with the physical aspects of fatigue. While its reliability has been established for various conditions, it has not been specifically examined in the context of MS [46, 47]. The Fatigue Severity Scale is a widely used tool in both clinical and research settings to measure the severity of fatigue and identify distinguishing features between two chronic medical disorders [48]. All of the included studies utilized Patient-reported outcomes, which can enhance the accuracy and reliability of data, facilitating the derivation of significant conclusions from various research findings..

The mechanism underlying postural instability in individuals with MS is a multifaceted process involving various factors. These factors include impaired lower leg muscle function [28], compromised PC [31], and an increased risk of falls [29]. Fatigue in MS patients is often associated with motor exertion, which can lead to decreased performance on balance scales and increased postural sway [27, 30]. Gender differences were inconclusive in the context of a fatiguing task’s impact on PC in individuals with MS [27]. In addition to task complexity, vision, and symptomatic fatigue [31], another mechanism that contributes to postural instability in MS patients is the sensorimotor mechanism. Fatigue affects the performance of individuals with MS on the Berg Balance Score [29], a measure of balance. Maintaining balance relies on the integration of sensorimotor information [49], which involves combining sensory input with motor output for coordinated movement [50]. Also, muscle fatigue can disrupt the central perception system, leading to a lack of motor control and an increased risk of falls. Individuals with MS often have deficient sensory systems and rely more on vision to maintain their postural balance [31]. In this regard, CNS lesions in MS can impact sensory and motor function, leading to sensory loss and fatigue, which may contribute to poor balance control [31]. Moreover, in the context of the sensorimotor mechanism, lower leg muscle function becomes a crucial target for intervention to improve gait, balance, and fall risk among individuals with MS [28]. This is because muscle fatigue can be divided into central and peripheral components, with central fatigue originating in the CNS and peripheral fatigue occurring at or distal to the neuromuscular junction [51, 52]. Individuals with MS experience a greater level of peripheral muscle fatigue while walking compared to those who are healthy [53]. This may be why walking tests, such as the 6MWT, are commonly used in MS research to assess walking fatigue due to their reliability and validity [54]. Therefore, it appears that the primary issue is related to peripheral muscle fatigue. Understanding these mechanisms is crucial for developing effective interventions to improve postural stability and reduce the risk of falls in individuals with MS.

The strength of this systematic review lies in its adherence to the PRISMA guidelines, which ensure the use of optimal methods for conducting and reporting systematic reviews. However, there are some limitations to consider. The heterogeneity in disability levels and progression among individuals with MS can introduce a significant risk of bias. This is because the included studies may not adequately represent the entire MS population, particularly in terms of disability severity and progression. Another limitation of the studies included in the investigation is that they utilized different drugs, such as antifatigue drugs [29, 31] or fampridine [30]. This variation in medication could have influenced the severity of fatigue or the walking speed, resulting in inconsistent outcomes in terms of PC in individuals with MS who are dealing with fatigue. Furthermore, the fair methodological quality of the included studies may also increase the risk of bias, as these studies may have limitations that affect the reliability and validity of their findings.

## Conclusion

Studies have shown that people with MS may struggle with balance due to fatigue, and 6MWT is commonly used to assess fatigue-related factors. However, more research is needed to account for confounding variables such as disability levels, disease progression, and medication use. To address this, researchers can use statistical techniques, match participants based on specific characteristics, or conduct longitudinal studies. By implementing these strategies, future studies can provide more accurate and reliable results, leading to a better understanding of the impact of fatigue on PC in individuals with MS.

## Data Availability

Data are available on request from the corresponding author..

## Abbreviations

CNS: Central Nervous System
MS: Multiple Sclerosis
PC: Postural Control
